# Comparison of two frailty screening tools in older patients with colorectal cancer

**DOI:** 10.1186/s12877-023-03974-3

**Published:** 2023-05-15

**Authors:** Han Zhao, Xinlin Lu, Senshuang Zheng, Danmei Wei, Lizhong Zhao, Yuan Wang, Geertruida H. de Bock, Wenli Lu

**Affiliations:** 1 Department of Epidemiology and Statistics, School of Public Health, Tianjin Medical University, Tianjin, China; 2 University Medical Center Groningen, University of Groningen, Groningen, The Netherlands; 3 Department of Gastroenterology, Tianjin Union Medical Center, Tianjin, China; 4 22 Qixiangtai Road, Tianjin, 300070 China

**Keywords:** Frailty, Geriatric Assessment, Geriatric 8, Korean Cancer Study Group Geriatric score, Colorectal cancer

## Abstract

**Introduction:**

Geriatric assessment (GA) is widely used to detect vulnerability in older patients. As this process is time-consuming, prescreening tools have been developed to identify patients at risk for frailty. We aimed to assess whether the Geriatric 8 (G8) or the Korean Cancer Study Group Geriatric Score (KG-7) shows better performance in identifying patients who are in need of full GA.

**Materials and methods:**

A consecutive series of patients aged ≥ 60 years with colorectal cancer were included. The sensitivity, specificity, predictive value, and 95% confidence intervals (95% CI) were calculated for the G8 and the KG-7 using the results of GA as the reference standard. ROC(Receiver Operating Characteristic) was used to evaluate the accuracy of the G8 and the KG-7.

**Results:**

One hundred four patients were enrolled. A total of 40.4% of patients were frail according to GA, and 42.3% and 50.0% of patients were frail based on the G8 and the KG-7, respectively. The sensitivity and specificity of the G8 were 90.5% (95% CI: 77.4–97.3%) and 90.3% (95% CI: 80.1–96.4%), respectively. For the KG-7, the sensitivity and specificity were 83.3% (95% CI: 68.6–93.0%) and 72.6% (95% CI: 59.8–83.1%), respectively. Compared to the KG-7, the G8 had a higher predictive accuracy (AUC: (95% CI): 0.90 (0.83–0.95) vs. 0.78 (0.69–0.85); *p* < 0.01). By applying the G8 and the KG-7, 60 and 52 patients would not need a GA assessment, respectively.

**Conclusion:**

Both the G8 and the KG-7 showed a great ability to detect frailty in older patients with colorectal cancer. In this population, compared to the KG-7, the G8 had a better performance in identifying those in need of a full Geriatric Assessment.

## Introduction

Frailty is a clinical state that increases the likelihood of adverse health outcomes and deterioration in the physiological capacity of several organ systems [1, 2]. Frailty accelerates the decrease in physiological reserve, thus increasing susceptibility to [3]. When faced with the same stressful events, frail individuals have more severe functional loss and are less likely to complete recovery than individuals without [2]. The prevalence of frailty increases with age. With the increase in global ageing, frailty has become a hot spot in the field of geriatrics in recent years.

The incidence of frailty in older cancer patients is significantly higher than that in people of the same age [4], as both cancer itself and the treatments might be significant additional stressors that challenge the physiological reserves of patients [5]. Colorectal cancer remains the most commonly diagnosed cancer among older persons globally, and surgery is often the recommended [6]. Due to the increased focus on value-based care, some older patients may prefer a better quality of life rather than sacrificing well-being for the possibility of more prolonged [7]. Current treatment plans in cancer care are based on less evidence and heterogeneity in the ageing process. However, chronological age does not describe this well. Frailty assessments can detect more health problems, prevent function deterioration, and determine the most feasible cancer [8]. For patients undergoing surgery, a frailty assessment can help predict whether the patient will benefit from surgery and tolerate the inherent iatrogenic [9]. Frailty assessment is also vital in deciding if a patient could benefit from the proposed [5, 10]. Therefore, it is necessary to assess the frailty degree of older patients with cancer to optimize personalized care strategies.

Geriatric assessment (GA) is one of the most widely used frailty assessments in cancer patients. GA is a complex and time-consuming process. Hence, some researchers have proposed a two-step approach, which includes using a simpler screening tool first to identify patients who might benefit from the [11]. There are currently several frailty screening tools for older patients, including the Groningen Frailty Indicator (GFI), the Vulnerable Elders Survey-13 (VES-13), the Geriatric 8 (G8), and the Korean Cancer Study Group Geriatric Score (KG-7)[12]. The GFI is a frailty self-assessment tool with good psychometric [13], and the VES-13 was developed to screen community-dwelling older people who are at risk of functional [14–16]. Both the G8 and the KG-7 were designed to screen for frailty in older patients with cancer. The KG-7 is a novel geriatric screening tool. Shorter screening tools are able to identify patients who might benefit from a full GA. In this study, the G8 and the KG-7 were compared as screening tools for detecting frailty in older patients with colorectal cancer based on a reference text of ≥ 2 deficits in GA.

## Materials and methods

### Participants

The patients were recruited from the Outpatient and Inpatient Department of Agroecology, Tianjin People’s Hospital, from October 2020 to April 2021. Patients were enrolled in a consecutive manner based on criteria. The inclusion criteria were being 60 years or older, being newly diagnosed with colorectal cancer, and being able to understand and communicate in Chinese. The exclusion criteria were having severe dementia, symptoms of brain metastasis, or serious neurological and psychiatric problems. The medical students went to the department office to determine with the doctors which patients met the criteria and summarized the information of these patients. All patients were informed about the study and gave written consent. The study was approved by the Ethics and Clinical Research Committee of Tianjin Medical University, which confirmed that the study followed the Declaration of Helsinki.

We use the equation below to calculate the sample size:

1 $$N = U^{2}_{1 - \alpha /2} {P_0}\left( {1 - {P_0}} \right)/{d^2}$$

where P_0_ represents the prevalence of frailty, which we set as 42%[17], ɑ represents the accepted small probability of a false-positive result, which is 0.05 in this study, and d represents the admissible error. Thus, the sample size was calculated to be 121, and the final sample size was estimated to be 146 by considering a 20% rate of no-response and lost visit cases. However, due to the managed lockdown and the decreasing number of inpatients during COVID-19, 115 patients were finally enrolled.

### Data collection

Investigators, including doctors, nurses from relevant departments, and medical graduate students, were trained through meetings to master the survey content and process. Participants were surveyed after admission to the hospital and before surgery. General information and disease-related information were collected by medical students from an integrated medical record system. The MCIRS-G was assessed with the assistance of a doctor. The results of the ADL, IADL, MNA GDS-15, MOSS-SSS, and two screening tools were assessed at the bedside by medical students through face-to-face interviews of patients and families. Telephone surveys were used as a supplement for face-to-face interviews during the COVID-19 pandemic. Nurses measured the TUG, MMSE, mid-arm circumference, and calf circumference.

### Geriatric assessment (GA)

GA was performed by several reliable and valid tools to evaluate seven clinical domains with different cut-off values (Table 1). The function was assessed by the Barthel index and Lawton-Brody Instrumental Activity of Daily [18, 19]. The time up and go test (TUG) was used to assess [20]. The nutrition domain was evaluated using the Mini Nutritional Assessment questionnaire (MNA)[21]. Comorbidity was measured using the Modified Cumulative Illness Rating Scale (MCIRS-G)[22, 23]. The Mini-Mental State Examination (MMSE) was used to evaluate cognitive [24]. The Geriatric Depression Scale-15 (GDS-15) was used to assess [25]. The Medical Outcomes Study Social Support Survey (MOS-SSS) was used to evaluate social [26, 27]. The number of medications was used to assess [28]. We defined patients with ≥ 2 deficits in GA as vulnerable based on the impairment cut-off score, as was done in prior studies (see Table 1)[14, 29].

### Frailty screening tools

The G8 questionnaire was proposed by Bellera et al. in 2012 in a regional multicenter prospective [30, 31]. The G8 questionnaire consists of 8 items, including seven questions from the Mini Nutritional Assessment (MNA) and age, which are divided into three categories (< 80, 80–85, > 85). Seven questions from the MNA focus on food intake, weight loss, mobility, psychological status, body mass index, number of medications, and self-perception of health. The 8 items provide a total score ranging from 0 to 17 (no impairment). A score of ≤ 14 indicates frailty.

The KG-7 was recently developed as a screening tool for older patients to select who could benefit from complete [32]. The KG-7 is composed of 7 easy questions originating from GA distributed across different scales, including “bathing and showering” and “ascending stairs” (ADL), “shopping” (IADL), “self-view of nutritional status” and “number of medications” (MNA), “orientation of time and place” (MMSE) and “decline in interest” (GDS). Each question was answered “yes” or “no” with a total score ranging from 0 (heavily impaired) to 7 (no impairment). For “number of medications” and “decline in interest”, the negative answer was scored one point, and the positive answer was not scored, while for the remaining questions, the positive answer was scored [33]. Patients with a KG-7 score ≤ 5 should be regarded as frail.

**Table 1:** Cut-off value for different tests used

Test	Domain	Number of items	Range	Cut-off score
G8	Screening tool	8	0–17	≤ 14
KG-7	Screening tool	7	0–7	≤ 5
Barthel index	Function	10	0-100	≤ 95
IADL	Function	8	8–32	> 8
TUG	Mobility	1	-	≥ 12
MNA	Nutrition	18	0–30	≤ 23.5
MCIRS-G	Comorbidity	14	0–56	> 14
MMSE	Cognition	10	0–30	< 24
GDS-15	Depression	15	0–15	≥ 8
MOS-SSS	Social support	19	0–5	< 4
Number of medications	Polypharmacy	1	0–∞	> 4

### Statistical analysis

The sensitivity, specificity, positive predictive value (PPV), negative predictive value (NPV), and 95% confidence intervals (95% CI) of the screening tests were calculated using the original cut-off value from the literature. We used receiver operating characteristic (ROC) curve analysis to evaluate the diagnostic performance of the G8 and the KG-7 for the frail individuals. The DeLong test was used for the comparison of the two ROC curves[34]. Sensitivity and specificity according to different cut-off values were calculated and compared with the originals to establish the optimal cut-off points, which were determined by Youden’s index. In all analyses, the significance level was set at 5%. Data were analyzed using SAS 9.4 for Windows. ROC analyses were performed using MedClac software.

## Results

### Characteristics of patients

**Fig. 1:**
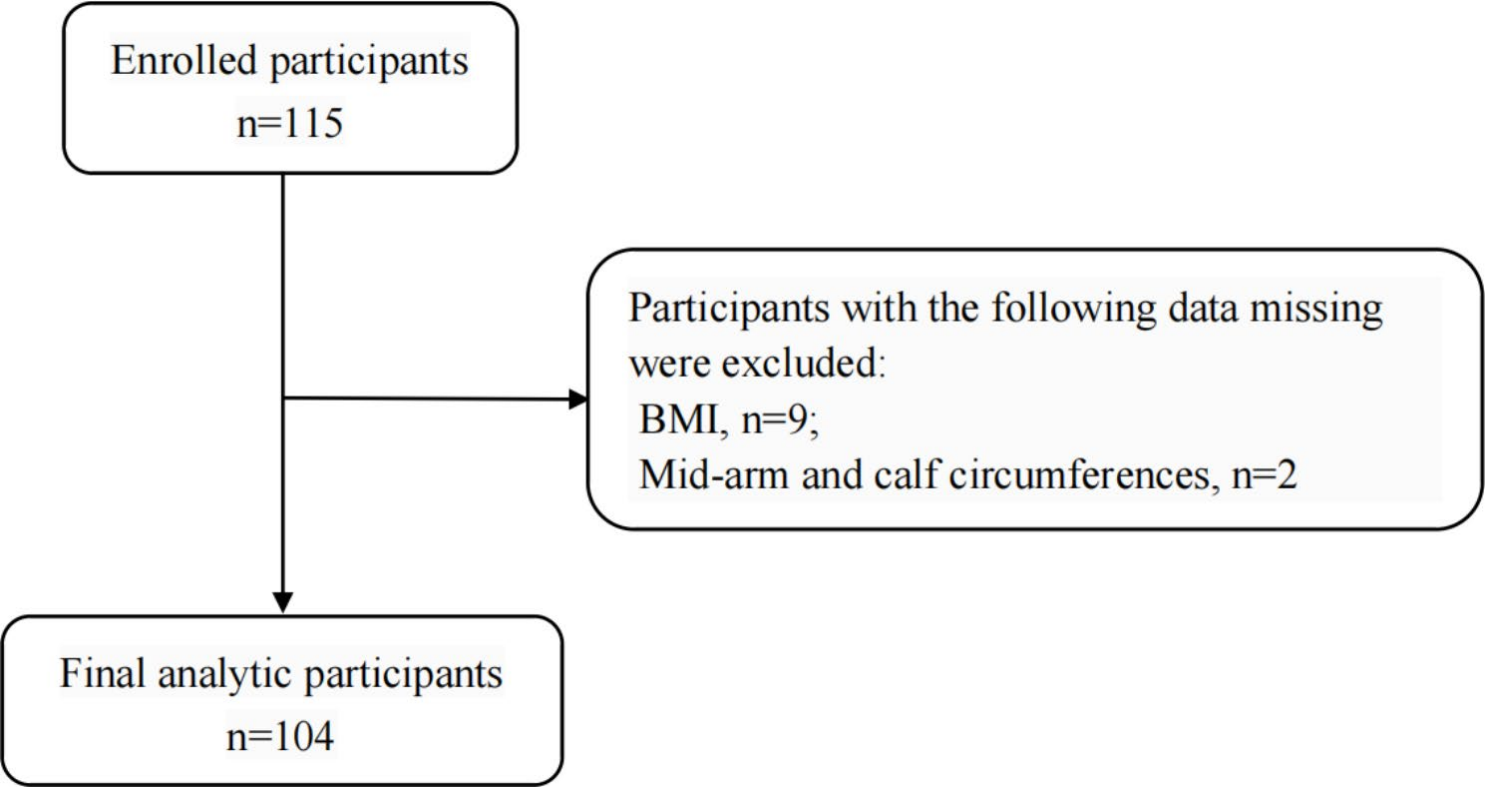
Flow chart of analytic sample

A total of 115 patients meeting the criteria were enrolled. Among them, nine people lacked BMI, and two lacked mid-arm circumference and calf circumference. One hundred and four patients were finally included in the study. There were no missing data for the G8, KG-7 items, or GA findings among 104 patients (Fig. 1). Few data were missing for the tumour characteristics: 17.3% in the Dukes stage and 11.5% in distant metastasis. The mean age was 68.7 ± 6.9 years old, and 62.5% of patients were males. More than half of the patients were aged between 60 and 70. In addition, the majority were married (97.1%), had an education level of less than high school (74.1%), had 56 patients (53.9%) in Dukes’ stage A or B and had nonmetastatic disease (83.7%)(Table 2).

**Table 2:** Demographic and clinical characteristics of the patients

Items		N	%
Mean age ($\overline {x} \pm s$)			68.7 ± 6.9
Age	60~	33	31.7
	65~	30	28.8
	70~	25	24.0
	75~	16	15.5
Sex	Male	65	62.5
	Female	39	37.5
Marital status	Married	101	97.1
	Widowed	3	2.9
Educational level	Less than high school	77	74.1
	High school or equivalent	17	16.3
	More than high school	10	9.6
Tumour location	Rectal	57	54.8
	Colon	46	44.2
Dukes stage	A	16	15.4
	B	40	38.5
	C\D	30	28.8
	Not know	18	17.3
Distant metastasis	Yes	5	4.8
	No	87	83.7
	Unclear	12	11.5

### GA results

Forty-two patients (40.4%) had two or more impairments assessed by GA. Sixteen patients had no impairment in GA, and six patients had four deficits in GA, which is the highest score. Patients with mobility field deficits are the most numerous, followed by the nutrition field. Patients with deficits in cognition or depression were the least common(Table 3).

**Table 3:** Impairment on the individual scales, Geriatric Assessment

Domain	N	%
Function	21	20.2
Mobility	68	65.4
Nutrition	47	45.2
Comorbidity	4	3.8
Cognition	3	2.9
Depression	3	2.9
Social support	12	11.5
Polypharmacy	21	20.2

### Results of the G8

According to the cut-off value of ≤ 14, 44 patients (42.3%) had an abnormal G8 result. The mean G8 score was 14.0 (SD: 2.3), 12.3 (SD: 2.1) for patients with abnormal GA, and 15.2 (SD: 1.4) for those with normal GA. Six patients were misidentify as frail by the G8, and they all had severe weight loss. Four patients misidentified as non-frail, and all had deficits in mobility (Table 4).

Using GA as the gold standard, the sensitivity and specificity were 90.5% (95% CI: 77.4–97.3%) and 90.3% (95% CI: 80.1–96.4%), respectively. The PPV was 86.4% (95% CI: 74.6–93.2%), and the NPV was 93.3% (95% CI: 84.6–97.3%). The best cut-off value for identifying frailty in our population was estimated at a G8 score ≤ 14 (Table 5).

**Table 4:** The results of two screening tools compared with GA (n%)

	G8			KG-7	
	≤14	>14		≤5	>5
abnormal GA	38(36.5)	4(3.9)		35(31.7)	7(6.7)
normal GA	6(5.7)	56(53.9)		17(16.4)	45(43.3)
Total	44(42.3)	60(57.7)		52(50.0)	52(50.0)

### Results of the KG-7

The KG-7 screened 52 patients (50%) as positive for complete GA. The mean KG-7 score was 5.3 (SD: 1.0), 5.8 (SD: 0.5) in patients with normal GA, and 4.6 (SD: 1.1) in those with abnormal GA. Only 5 people answered no to item 1(“Can you take a shower or bath without help?”), 7 people answered no to item 3(“Can you take care of all shopping needs independently?”), and all of the patients endorsed item 6(“What year, month, and day is this?”) (Table 4).

For the cut-off value ≤ 5, the sensitivity and specificity were 83.3% (95% CI: 68.6–93.0%) and 72.6% (95% CI: 59.8–83.1%), respectively, and the PPV and NPV were 67.3% (95% CI: 57.3–75.9%) and 86.5% (95% CI: 76.3–92.8%), respectively. In this study, the optimal cut-off value for identifying frail patients was estimated at a KG-7 score ≤ 5, consistent with previous studies’ results (Table 5).

**Table 5:** Diagnostic values for the G8 and KG-7 at different cut-off values

	cut-off value	Sensitivity, % (95% CI)	Specificity, % (95% CI)	PPV, % (95% CI)	NPV, % (95% CI)
G8	≤ 12	52.4 (36.4–68.0)	98.4 (91.3–100.0)	95.7 (75.5–99.4)	75.3 (68.9–80.8)
	≤ 13	61.9 (45.6–76.4)	91.9 (82.2–97.3)	83.9 (68.5–92.6)	78.1 (70.6–84.1)
	≤ 14	90.5 (77.4–97.3)	90.3 (80.1–96.4)	86.4 (74.6–93.2)	93.3 (84.6–97.3)
	≤ 15	95.2 (83.8–99.4)	46.8 (34.0-59.9)	54.8 (48.7–60.7)	93.5 (78.5–98.3)
KG-7	≤ 4	28.6 (15.7–44.6)	100.0 (94.2–100.0)	100.0 (-)	67.4 (63.1–71.4)
	≤ 5	83.3 (68.6–93.0)	72.6 (59.8–83.1)	67.3 (57.3–75.9)	86.5 (76.3–92.8)

Abbreviations: CI, confidence interval; PPV positive predictive value; NPV negative predictive value

### Comparisons of predictive accuracy

**Fig. 2:**
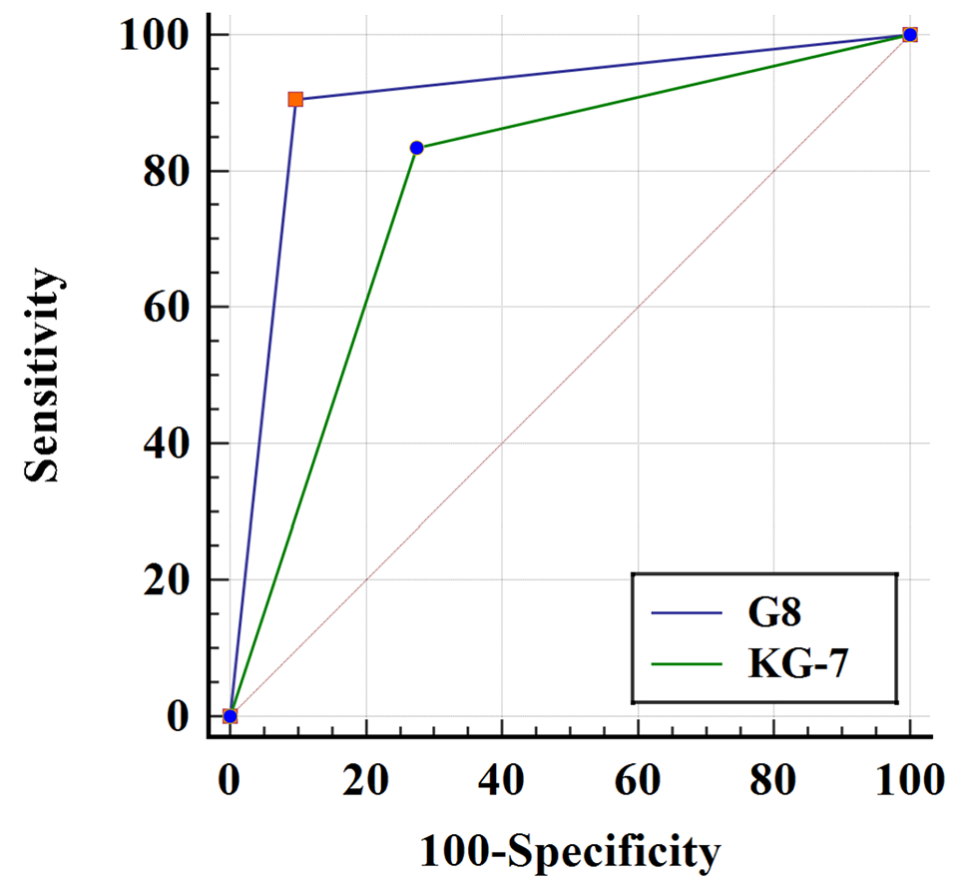
ROC curves of the G8 and the KG-7

As shown in Fig. 2, using GA as a reference standard, the AUCs of the G8 and the KG-7 were 0.90(95% CI: 0.83–0.95) and 0.78(95% CI: 0.69–0.85),respectively, indicating acceptable or good accuracy. ROC contrasts showed that the G8 had a significantly better ability than the KG-7 to distinguish patients with a normal GA and patients with an abnormal GA (*z* = 2.80; *p* < 0.05). Thus, the G8 is superior to the KG-7 for identifying patients who need a full GA. When using the G8 compared to the KG-7, fewer GAs would have been performed (42.3% instead of 50.0%).

## Discussion

To identify frailty in cancer patients, the International Society of Geriatric Oncology (SIOG) recommended using GA in patients with cancer. Since completing a GA is time-consuming, researchers have proposed a two-step approach that uses a pre-screening tool before the GA. In this study, we evaluated two screening tools’ capacity to identify older patients with colorectal cancer who were in need of accepting GA. The results showed that both the G8 and the KG-7 could differentiate patients who were positive for implementing GA. The G8 has higher accuracy in identifying frail patients. The best cut-off value of the G8 and the KG-7 were consistent with previous [30, 32].

In our sample, 40.4% had ≥ 2 impairments in GA. This result was close to similar studies with older patients with colorectal [35, 36]. Mobility and nutrition impairments have been detected in most patients. Mobility assessed the balance and gait speed of patients and was important in determining patients’ fitness for treatment. Older patients with colorectal cancer face many factors that deteriorate mobility that are observed in the general older population. In addition, there are disease-related disorders, including bowel dysfunction, pain, and [37]. Physical therapy, occupational therapy, and promoting physical activity were used as [38]. Emerging evidence proves that physical activity, which reduces the risk of mobility restrictions and increases independence, is beneficial for individuals with colorectal cancer, even at a low intensity [39]. The nutrition domain has been associated with the risk of malnutrition and indicates poorer nutrition and lower chemotherapy [40]. Adherence to an unhealthy lifestyle and dietary habits are risk factors for colorectal cancer incidence. Malnutrition has a high prevalence in colorectal cancer [41]. The effect of lower dietary intake, ageing, and the tumour resulted in a decline in nutrition status, which can lead to a complex malnutrition syndrome with an impact on [42]. Patients who are abnormal in nutrition should be referred to a nutritionist. According to a previous RCT, optimal nutrition management can improve frailty in older [43].

Compared to the KG-7, the G8 had higher sensitivity, specificity, and AUC, consistent with the previous [32]. According to the G8, fewer non-frail patients were regarded as needing full GA compared to the KG-7 (4 in the G8 vs. 7 in the KG-7), thus reducing the number of unnecessary GAs. Therefore, the G8 was superior for assessing older colorectal cancer patients. Several studies have validated the ability of the G8 to identify older cancer patients who may benefit from GA [29, 31]. Although the effectiveness of the G8 varied according to the tumour [31, 44], G8 maintains a high level of sensitivity to detect [45]. In previous studies, the superior sensitivity (77.0-98.0%) of the G8 was always expensing the specificity (60.0-91.0%), inducing a high number of false-[29, 46–48]. However, in this study, both the sensitivity and specificity were high. High sensitivity and specificity were also reported by Velghe et al.[49]. This may be caused by the dominance of the nutrition domain in the G8 and the result that nutrition was the second most impaired domain in this study.

The G8 showed oversensitive to malnutrition and under-sensitive to mobility. To further modify the G8, we propose to raise the score of the weight loss item (loss of weight during the last months) in the G8 to limit false-positives, which is consistent with Martinez-Tapia et al.[44]. Moreover, we also recommend enhancing mobility assessment to limit false-negatives using methods such as adding relevant items, an idea consistent with Petit-Monéger et al.[50]. The KG-7 shows a defect in its content, as certain items had few negative answers. We recommend changing the answer to the question to multiple options to improve the ability to distinguish between frail and non-frail individuals. Indeed, our opinion about improving the G8 and the KG-7 needs to be proven by more studies based on large populations.

The best cut-off values for the G8 and the KG-7 were 14 and 5, respectively. We contrasted the sensitivity and specificity of different cut-off values to find the optimal cut-off that can provide good sensitivity without excessively deteriorating the specificity. The best cut-off values for the G8 and the KG-7, as confirmed by ROC analysis in this study, were consistent with findings from previous [30, 32].

To our knowledge, this is the first study to evaluate the effectiveness of the G8 among Chinese cancer patients. Although we only applied the G8 in frailty screening in colorectal cancer patients, we hope this first attempt could provide insight into using the G8 in other types of cancer patients in China. We confirmed the effectiveness of two shorter screening tools, i.e., the G8 and KG-7, for identifying vulnerable populations. We tried to reduce the demand for GA since China has the most significant number of cases and a shortage of healthcare workers worldwide. The KG-7 was chosen for comparison to the G8 because it targets the Asian population. We aimed to confirm that the G8 could also be used among to Asians.

The results of this study should be interpreted with caution due to the following limitations. First, we faced some limitations related to COVID-19. Our evaluation was based on a small sample, as COVID-19 pandemic led to a lower volume of patients at the time of enrolment. If the period of enrolling participants had been extended, the study might have achieved greater statistical power. The lockdown impacted patients’ interactions with investigators, so telephone interviews were used as a supplement to face-to-face interviews. Telephone interviews might lead to a loss of visual and other nonverbal cues, but it is a suitable method considering available resources in such an extraordinary period. Second, the population we have finally included is relatively young. The young age of the study participants may influence the comparison between the G8 and KG-7. Older subjects have more mobility deficits, and the G8 has lower discrimination on this issue than the KG-7. The performance of the G8 might decrease in older subjects. Finally, fewer comorbidities and cognitive deficits in our population may be caused by volunteer bias. The number of comorbidities increases with age, and the younger population results in fewer comorbidity deficits. We excluded patients with severe mental illness, resulting in fewer cognitive deficits in our people. Thus, caution is indicated before generalizing results to the general population.

## Conclusion

In summary, we have offered evidence that both the G8 and the KG-7 have adequate abilities to identify frailty in older patients with colorectal cancer. The G8 has a better capacity to detect who should receive the GA in this population.

## Data Availability

The raw data used in this analysis are available from the corresponding author upon reasonable request.

## List of abbreviations

GA: Geriatric Assessment
ROC: Receiver Operating Characteristic
G8: Geriatric 8
KG-7: Korean Cancer Study Group Geriatric Score
GFI: Groningen Frailty Indicator
VES-13: Vulnerable Elders Survey-13
ADL: Activities of Daily Living
IADL: Instrumental Activities of Daily Living Scale
TUG: Time up and go test
MNA: Mini Nutritional Assessment questionnaire
MCIRS-G: Modified Cumulative Illness Rating Scale
MMSE: Mini-Mental State Examination
GDS-15: Geriatric depression Scale-15
MOS-SSS: Medical Outcomes Study Social Support Survey
CI: Confidence interval
PPV: Positive predictive value
NPV: Negative predictive value
SIOG: The International Society of Geriatric Oncology
RCT: randomized and controlled clinical trials
